# Experiences of Interdisciplinary Providers Deploying Telehealth During the COVID-19 Pandemic

**DOI:** 10.1093/geroni/igab046.2234

**Published:** 2021-12-17

**Authors:** Katelyn Moore, Zachary Hathaway, Gloria Gonzalez-Kruger, Kymberlee Montgomery, Rose Ann DiMaria-Ghalili, Martha Coates

**Affiliations:** 1 Drexel University, Philadelphia, Pennsylvania, United States; 2 Drexel University, College of Nursing and Health Professions, Philadelphia, Pennsylvania, United States; 3 Drexel University, Bryn Mawr, Pennsylvania, United States

## Abstract

The expansion of telehealth services during COVID-19 is critical for healthcare delivery. This study describes the facilitators and barriers experienced by providers integrating telehealth during COVID-19. The sample consisted of 441 interdisciplinary providers (RNs, APNs, PAs, DPTs, RDs, mental health counselors) who were faculty or alumni of a college of nursing and health professions and completed the online telehealth provider survey. 53% of respondents were nurses/APNs, 59% implemented telehealth within the first week of federal legislation, and 48% received telehealth training once the pandemic started. Respondents reported telehealth changed several services provided during the pandemic (e.g., increased prescription of longer-term medication refills, increased counseling sessions). The greatest reported barrier to utilizing telehealth during the pandemic was the older adults’ ability to utilize technology. Understanding the facilitators and barriers experienced by providers during COVID-19 will lead to more robust healthcare delivery models to enhance health outcomes in older adults.

